# Variations in S-100Β and Neuron-Specific Enolase Levels During Functional Endoscopic Sinus Surgery Under Moderately Controlled Hypotension Using Four Distinct Anesthetic Protocols: A Randomized Controlled Study

**DOI:** 10.3390/medicina62061006

**Published:** 2026-05-22

**Authors:** Sotiria Rizopoulou, Spyridon Lygeros, Anne-Lise de Lastic, Dimitra Georgakopoulou, Gerasimos Daniilidis, Athanasia Voulgary, Diamanto Aretha

**Affiliations:** 1 Department of Anesthesiology and Critical Care Medicine, General University Hospital of Patras, School of Medicine, University of Patras, 26504 Patras, Greece; med6451@upnet.gr (S.R.); athanasia13494@hotmail.com (A.V.); 2 Department of Otorhinolaryngology, General University Hospital of Patras, School of Medicine, University of Patras, 26504 Patras, Greece; slygeros@upatras.gr (S.L.); up1096481@upatras.gr (G.D.); 3 Laboratory of Immunohematology, Pathology Clinic, General University Hospital of Patras, School of Medicine, University of Patras, 26504 Patras, Greece; delastic@upatras.gr (A.-L.d.L.); up1019151@upnet.gr (D.G.)

**Keywords:** functional endoscopic sinus surgery, controlled hypotension, anesthesia techniques, sevoflurane, propofol, ketamine, magnesium sulfate, S-100Β, neuron-specific enolase, neuroprotection

## Abstract

*Background and Objectives*: Controlled hypotension during functional endoscopic sinus surgery (FESS) improves surgical field visibility but may pose a risk of subclinical cerebral hypoperfusion. Serum S100Β and neuron-specific enolase (NSE) are established biomarkers of glial and neuronal injury and may reflect perioperative neuroprotection associated with different anesthetic regimens. This study evaluated the effect of four anesthetic protocols on perioperative brain biomarker release during FESS. *Materials and Methods*: In this single-center, randomized, controlled trial, 88 adult patients (ASA I–III) undergoing FESS under moderately controlled hypotension (mean arterial pressure < 55 mmHg) were allocated to one of four groups: propofol–remifentanil, propofol–remifentanil with ketamine–magnesium, sevoflurane–remifentanil, or sevoflurane–remifentanil with ketamine–magnesium. Serum S100Β and NSE concentrations were measured at three timepoints: early intraoperatively, during hypotension, and at the end of surgery. Biomarker data were analyzed using nested ANOVA and linear mixed-effects models adjusted for relevant covariates. Secondary outcomes included recovery characteristics, surgical field quality, bleeding scores, and perioperative hemodynamics. *Results*: Baseline demographic and perioperative characteristics were comparable across groups. The group receiving sevoflurane–remifentanil combined with ketamine–magnesium showed the lowest S100B levels (*p* = 0.01 compared to the propofol–remifentanil group; *p* = 0.04 compared to the sevoflurane–remifentanil group). Additionally, NSE concentrations were markedly lower in both sevoflurane groups (sevoflurane–remifentanil and sevoflurane–remifentanil plus ketamine–magnesium) compared to the propofol–remifentanil group (*p* = 0.003 and *p* = 0.007, respectively). No intergroup differences were observed at baseline and surgical field quality, bleeding, and hemodynamic parameters did not differ significantly among groups. Recovery and extubation times were shortest with propofol–remifentanil, whereas ketamine–magnesium prolonged emergence. *Conclusions*: Anesthetic technique significantly influences perioperative brain biomarker release during FESS. Sevoflurane-based regimens, with or without ketamine–magnesium, demonstrate more favorable neurobiological profiles under controlled hypotension, although propofol-based anesthesia offers faster recovery.

## 1. Introduction

Functional endoscopic sinus surgery (FESS) is a minimally invasive technique used to treat chronic sinusitis. The procedure carries risks due to the proximity of vital structures like the orbit, brain, and carotid vessels. Furthermore, limited surgical exposure and challenges in operative field visualization, as well as intraoperative bleeding that may obscure endoscopic visualization, may increase the chances of complications such as optic nerve injury, dural puncture, or hemorrhage [1]. For these reasons, inducing hypotension during surgery is essential. A bloodless surgical field provides better visibility of the operative field, with reduced risk of injury to adjoining structures [2,3]. On the other hand, several studies have reported a significant correlation between intraoperative hypotension and organ injury [4,5,6].

A variety of pharmacological agents have demonstrated efficacy in achieving controlled hypotension to enhance surgical outcomes [2,3,7,8,9]. There is no definitive evidence indicating that either propofol or sevoflurane is superior for brain protection across all surgical contexts; each agent offers distinct advantages, and current research findings remain mixed and dependent on the specific clinical scenario [10,11,12,13]. Ketamine was found to decrease spreading depolarizations, which are associated with secondary brain injury, suggesting a potential neuroprotective role in cases of traumatic brain injury [14,15,16,17]. Animal and experimental studies on traumatic brain injury show that magnesium given before or soon after injury may reduce neuronal damage and improve motor or behavioral outcomes, indicating a neuroprotective effect [18,19,20,21].

Prompt recognition and evaluation of perioperative neurological dysfunction in patients is crucial in clinical practice. Biomarkers are biological molecules that function as indicators for the initiation or progression of biological processes, or as metrics for assessing therapeutic outcomes. Within this context, biomarkers have emerged as important supplementary tools to conventional diagnostic approaches, including electroencephalography, sensory and motor evoked potentials, transcranial Doppler ultrasound, near-infrared spectroscopy, and a range of imaging methods [22]. Non-traumatic ischemic injuries result from oxygen deprivation during certain surgeries, extracorporeal membrane oxygenation, acute stroke, or cardiac arrest [22]. Numerous studies have aimed to identify specific biochemical markers that can indicate ischemic brain injuries in these conditions [22,23,24,25,26,27,28]. The presence of these biomarkers may also be observed in conditions unrelated to ischemic injuries; for instance, S100B and neuron-specific enolase (NSE) can be elevated in cases of infections and sepsis [29], which may be associated with tumors [28,30].

An ideal biomarker for the diagnosis, prognosis, and monitoring of brain injury should exhibit specificity to the central nervous system, be easily obtainable, and demonstrate resilience against cytoplasmic and extracellular proteolytic degradation. Furthermore, it must provide high diagnostic specificity and sensitivity, along with predictive value for both short-term and long-term outcomes. Additionally, it is essential that biomarker concentrations accurately correspond to the degree of CNS injury and respond favorably to neuroprotective interventions. Despite the confirmed correlations between the changes in biochemical markers and brain injuries, the use of individual markers is limited due to their low sensitivity and specificity [31]. S100B and NSE are considered the most promising and extensively studied biomarkers for brain injury [32,33]. S100B is a calcium-binding protein released into the extracellular space after cell injury, either actively or passively. While typically found in low levels in serum or cerebrospinal fluid, its concentration rises significantly during intracranial injuries [21,31]. NSE is an isoenzyme of enolase (EC 4.2.1.11), which can be primarily found in the cytoplasm of neurons. It is not normally secreted from cells; it is released only in the event of neuronal damage, so it is considered to be a biomarker that directly assesses functional neuronal damage [22,29,34,35]. This study compares four pharmacological anesthetic protocols by measuring S100Β and NSE as indicators of brain injury, along with surgeon satisfaction [30] and surgery duration.

## 2. Materials and Methods

### 2.1. Study Design and Patient Selection

This study comprises a single-center, randomized, comparative, controlled clinical trial with concealed allocation in a 1:1:1:1 ratio, conducted at the General University Hospital of Patras, a tertiary referral hospital in Patras, Greece. The study protocol received approval from our institution’s Ethics Committee (institutional code: 509/2024). Additionally, it was prospectively registered with the Clinical Research Information Service under registration number NCT07181564 preceding the recruitment of the initial participant. Written informed consent was obtained from parents or legal guardians before any participant was enrolled in the study. The research was conducted in accordance with Good Clinical Practice guidelines and adhered to the principles outlined in the Declaration of Helsinki [36].

The trial included patients 18 years of age or older, who were classified as American Society of Anesthesiologists Physical Status (ASA-PS) I to III. The study included healthy patients classified as ASA-PS I, those with mild systemic diseases such as current smokers, social alcohol drinkers, or mild obesity classified as ASA-PS II, and patients with morbid obesity (BMI > 40) classified as ASA-PS III. Patients were excluded if they had conditions precluding controlled hypotension, such as pregnancy, hypertension, coronary heart disease, heart failure, stroke, asthma, diabetes, known allergies or contraindications to study drugs or anesthetics, impaired kidney function, or liver disorders. Given the potential adverse effects of magnesium, especially its neuromuscular and sedative properties, we excluded patients who may be at heightened risk for magnesium-related complications. This included individuals with significant renal impairment, due to decreased magnesium clearance, as well as those with known neuromuscular disorders such as myasthenia gravis. All participants were scheduled to undergo FESS under general anesthesia with controlled hypotension.

### 2.2. Randomization and Group Allocation

Participants were allocated to one of four anesthesia protocol groups using random permuted block randomization, which was implemented prior to the induction of anesthesia. All patients received propofol (2 mg/kg) and remifentanil (2 μg/kg), dosed according to Ideal Body Weight, for anesthesia induction. Subsequent anesthesia maintenance was conducted in accordance with each patient’s assigned study group. The maintenance protocols for the four groups were as follows: Group 1 received propofol/remifentanil; Group 2 received propofol/remifentanil with the addition of magnesium sulfate (Mg^+^) and ketamine (Mg^+^/ketamine); Group 3 received sevoflurane/remifentanil; and Group 4 received sevoflurane/remifentanil with Mg^+^/ketamine. Patients in Groups 2 and 4 received a 0.2 mg/kg IV ketamine bolus after anesthesia induction and then 0.15 mg/kg/h through infusion. They also received a 50 mg/kg IV magnesium sulfate bolus over 15 min, followed by an 8 mg/kg/h infusion until extubation. All patients were given 8 mg of intravenous dexamethasone during surgery to reduce airway swelling and minimize coughing upon extubation. Additionally, before extubation, each patient received an extra 20–60 mg of intravenous xylocaine to prevent coughing and complications such as bronchospasm, laryngospasm, asthma attacks, agitation, and tachycardia.

The group designation was kept confidential from patients, their legal guardians, attending nurses in the post-anesthesia care unit (PACU), and postoperative outcome assessors. However, the attending anesthesiologists and operating room staff were not blinded to the group assignments. Despite this, the investigators responsible for performing the statistical analysis of the data, as well as the individual measuring the levels of biomarkers, remained blinded to the group designations.

### 2.3. Perioperative Monitoring and Procedures

The study was conducted in the operating rooms of the General University Hospital of Patras between June 2025 and December 2025. Each surgical procedure was attended by two anesthesiologists and a nurse from the anesthesiology department. Upon arrival in the operating room, intravenous access was established in all patients and standard monitoring was initiated. Heart rate (HR) and mean arterial pressure (MAP) were recorded at predefined intraoperative timepoints. For blood sampling and invasive blood pressure monitoring, an arterial catheter was inserted into the radial artery under local anesthesia. Continuous invasive monitoring allowed for the maintenance of intraoperative hypotension, targeting a MAP of less than 55 mmHg. Blood gas analyses were performed to optimize arterial oxygen (PO_2_) and carbon dioxide (PCO_2_) levels during mechanical ventilation. The depth of anesthesia was assessed in all four groups utilizing bispectral index (BIS) technology (software version 3.50, Medtronic, Dublin, Ireland).

Anesthetic depth and hemodynamic targets were achieved through stepwise titration of anesthetic agents according to predefined clinical targets. BIS values were maintained between 35 and 40 under stable conditions. If the target MAP (<55 mmHg) was not achieved, anesthetic depth was increased incrementally through adjustments in propofol infusion or sevoflurane concentration [target range approximately 0.8–1.2 Minimum Alveolar Concentration (MAC)]. In instances where hypotension was not achieved, deeper anesthesia (BIS down to approximately 25) was temporarily permitted as part of a controlled titration strategy, prioritizing hemodynamic targets while maintaining patient safety. In cases where hypotension remained insufficient despite adequate depth (BIS ≤ 35), remifentanil infusion was increased up to a maximum of 750 μg/h. If further control was required, clonidine was administered as a rescue agent at the discretion of the anesthesiologist. Patients who experienced difficulty in achieving hypotension were excluded from the analysis; all cases included were managed successfully according to the established protocol. Continuous adjustments were made based on real-time monitoring of mean arterial MAP and BIS, ensuring appropriate anesthesia and maintained controlled hypotension.

Adverse effects potentially related to magnesium administration were prospectively monitored throughout the intraoperative period. Patients were assessed for clinical signs of magnesium-related effects, including excessive hypotension, bradycardia, or evidence of prolonged neuromuscular blockade. When prolonged neuromuscular blockade was suspected, neuromuscular recovery was closely monitored and managed according to standard clinical practice. Participants were not excluded solely on the basis of transient hemodynamic variations; however, predefined safety thresholds were applied to guide intraoperative management and ensure patient safety. No magnesium-related adverse events requiring protocol deviation or participant exclusion were observed during the study period.

### 2.4. Anesthesia Induction and Sample Collection Procedures

Anesthesia was induced with propofol at a dose of 2 mg/kg, remifentanil at 2 μg/kg, and rocuronium at 0.6 mg/kg. Following induction, endotracheal intubation was performed and mechanical ventilation was initiated. The ventilation parameters were adjusted to target an end-tidal carbon dioxide (ETCO_2_) level of 30–35 mmHg or a blood partial pressure of carbon dioxide (PCO_2_) of approximately 35 cmH_2_O. MAC values were recorded for sevoflurane. Blood gas analysis and samples for S-100Β and NSE were collected at predetermined intervals: 10 min after the onset of surgery, 20 min after the initiation of moderate controlled hypotension, and upon completion of the procedure. For the purpose of this study, moderate controlled hypotension was defined as a target MAP below 55 mmHg. Controlled hypotension is generally described in the literature as a deliberate reduction of MAP to approximately 50–65 mmHg or a 20–30% decrease from baseline values in order to reduce intraoperative bleeding and improve surgical field visibility [36]. Consistent with this widely accepted range, the MAP threshold applied in the present protocol represents the lower limit of this spectrum and was therefore considered a moderately aggressive yet clinically acceptable target. In the context of FESS, similar MAP targets (typically ranging between 50 and 65 mmHg) have been extensively used and are associated with improved operative conditions without compromising hemodynamic stability in appropriately selected patients [37,38]. Therefore, a MAP < 55 mmHg was selected as an operational definition of moderate controlled hypotension, balancing surgical requirements with patient safety.

After collection, the blood samples were allowed to thicken for 30 min at room temperature. Subsequently, they were centrifuged for 15 min at 1000× *g* to separate the serum, which was then stored at −80 °C for preservation. Access to the collected samples was restricted to two primary researchers. All samples were retained for a period of two years to ensure proper study documentation and potential future analysis.

### 2.5. Statistical Analysis and Calculation of Patient Sample

The sample size was determined using power analysis (G-power, F-tests, ANOVA: repeated measures, and within–between interactions). To detect a potential statistical difference among the four patient groups, considering three biomarker measurement timepoints, with α = 0.05, power = 90%, and an effect size of 0.25, a total of 80 patients was required—20 per group. To ensure adequate power after randomization and potential attrition, we conservatively enrolled 26 patients per group.

Data were analyzed using SPSS (version 27.0) and GraphPad Prism (version 10.6.1). The Kolmogorov–Smirnov and Shapiro–Wilk tests were used to assess normality. Normally distributed data were compared with mixed ANOVA and post hoc Student’s t-tests, applying Sidak’s correction for multiple comparisons. Greenhouse–Geisser’s epsilon correction was used to adjust for violations in sphericity. Non-normally distributed data were analyzed using the Kruskal–Wallis test and post hoc Mann–Whitney U-tests, with Dunn’s test adjustment. Chi-square tests were used for analog and categorical data.

Serum S100B and NSE were measured at three timepoints for each patient across the four anesthesia groups. Values below the limit of detection (LOD) were recorded as LOD/√2 and then all values were subsequently log-transformed. A nested one-way ANOVA group analysis, supplemented by Tukey’s multiple comparison test, was employed to identify differences in biomarkers across the groups. Linear mixed-effects models were also used, incorporating random intercepts for patients and an unstructured within-subject covariance for each timepoint. Both group and timepoint were included as fixed effects. The adjusted model also controlled for gender, smoking status, and weight. Restricted Maximum Likelihood (REML) estimation with Satterthwaite’s degrees of freedom was applied; comparisons of Estimated Marginal Means (EMMeans) were adjusted using the Sidak method. If no significant group × timepoint interaction was present, only the main effects were reported. Statistical significance was determined at *p* < 0.05.

### 2.6. Outcome Measures

The principal outcomes evaluated in this study were the changes in plasma S-100Β and NSE concentrations at three distinct timepoints: T1 (10 min following the onset of surgery), T2 (20 min after the initiation of controlled hypotension), and T3 (upon completion of the procedure). Since FESS is a relatively short procedure, the chosen timepoints were appropriate for tracking biomarker alterations. The T1 measurement was designated as the baseline, given that hypotension had not yet been observed at this timepoint (10 min after the initiation of surgery). The research examined variations in plasma biomarkers relative to the anesthetic pharmacological protocols employed. Secondary endpoints encompassed surgeon satisfaction measured using Likert scale ratings [30], bleeding score (Grade 1 to 5), recovery duration, Riker Sedation–Agitation Scale (SAS), Aldrete Score, perioperative hemodynamic parameters, drug doses, total operative time, and adverse events (delirium, cough, apnea, and nausea/vomiting). Throughout the duration of the study, the composition of the surgical team was maintained without change.

## 3. Results

### 3.1. Demographics and Baseline Characteristics

**Figure 1:**
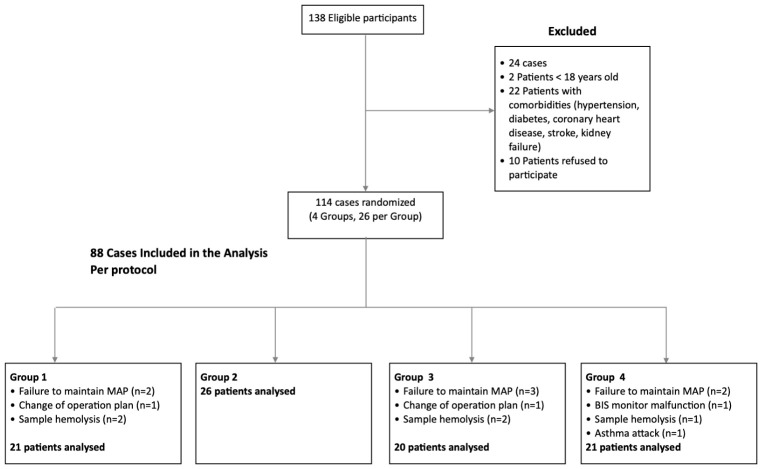
Study flowchart.

A total of 88 patients were included in the analysis (Figure 1). Baseline demographic and perioperative characteristics were comparable across the four anesthetic groups (Table 1). There were no statistically significant differences in gender distribution (*p* = 0.64), age (*p* = 0.85), weight (*p* = 0.60), or BMI (*p* = 0.43). ASA-PS was similarly balanced (*p* = 0.37), with most patients classified as ASA-PS I–II. Smoking status also did not differ significantly between groups (*p* = 0.20), while surgical duration was comparable across the four groups (*p* = 0.43), indicating similar procedural complexity and exposure time. Overall, the absence of statistically significant differences in demographic and perioperative variables confirms that the study groups were well balanced at baseline, minimizing potential confounding and strengthening the validity of subsequent comparisons in biomarker levels (S100B and NSE).

### 3.2. Primary Outcomes

#### 3.2.1. S100B Analysis

S100B was assessed at three different timepoints for each group. Specifically, 63 samples were collected from Group 1 (21 participants), 84 from Group 2 (26 participants), 57 from Group 3 (20 participants), and 69 from Group 4 (21 participants).

**Figure 2:**
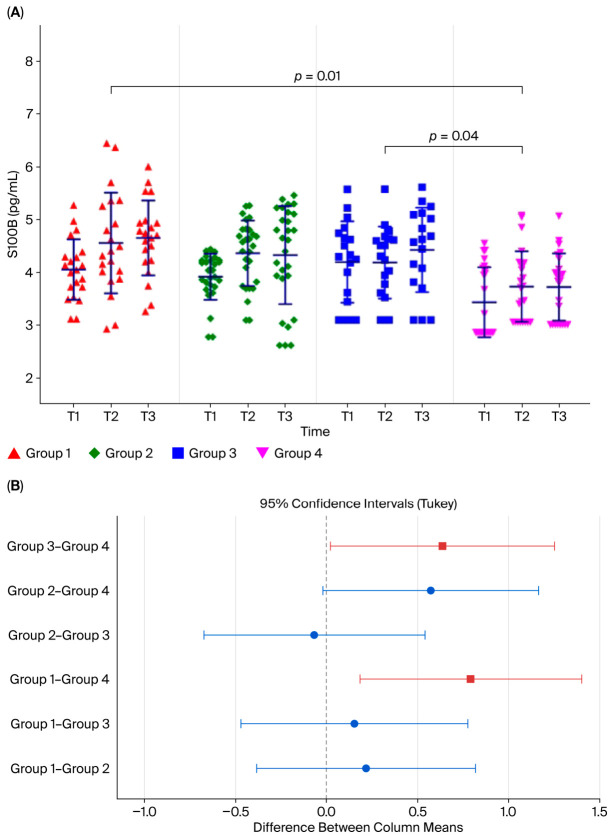
Mean (SD) S100B levels (**A**) and differences between group means (**B**). Statistically significant differences are presented as red squares (**B**). Group 1: propofol/remifentanil; Group 2: propofol/remifentanil with Mg^+^/ketamine; Group 3: sevoflurane/remifentanil; and Group 4: sevoflurane/remifentanil with Mg^+^/ketamine. T1: 10 min following the onset of surgery; T2: 20 min after the initiation of controlled hypotension; and T3: upon completion of the procedure.

A nested one-way ANOVA group analysis (Table 2 and Figure 2), using Tukey’s multiple comparison test, demonstrated that Group 4 exhibited the lowest ln(S100Β) values [Mean (SD): 3.63 (0.50)], with statistically significant reductions compared to Group 1 [Mean (SD), 4.42 (0.66), *p* = 0.01] and Group 3 [Mean (SD): 4.27 (0.70), *p* = 0.04), but not statistically significant differences demonstrated compared to Group 2 [Mean (SD): 4.20 (0.58), *p* = 0.06]. Further analysis employing a mixed-effects model (REML) with post hoc pairwise comparisons adjusted using the Sidak method showed that Group 1 had significantly higher ln S100B levels than Group 4 at T3 (mean difference: Δln = 0.91, *p* = 0.001). Additionally, Group 2 presented significantly elevated ln S100B levels relative to Group 4 at T2 (*p* = 0.03, mean difference: Δln = 0.69) and at T3 (*p* < 0.001, mean difference: Δln = 0.84). No statistically significant differences were observed between groups at T1 (ln S100B baseline level; see Figure S1). Overall, Group 4 consistently had the lowest S100B measurements throughout each phase.

Serum S100B concentrations were also analyzed on a logarithmic scale employing a multiple linear regression model. After adjusting for gender, smoking status, and body weight, the analyses demonstrated a significant main effect of group on ln S100B (F(3,80) = 3.29, *p* = 0.025), as well as a significant main effect of timepoint (F(2,86) = 3.96, *p* = 0.022). The interaction between group and timepoint was not statistically significant (F(6,85) = 0.704, *p* = 0.65), indicating that the temporal patterns remained consistent across different anesthetic protocols. Regarding other covariates, female sex (*p* = 0.034) and smoking status (*p* = 0.03) were independently associated with elevated ln S100B levels, whereas increased body weight was associated with reduced concentrations (*p* = 0.008). An evaluation of model residuals confirmed an appropriate model fit and no evidence of distributional violations. Specifically, female sex was linked to a 44% rise in S100B levels (*p* = 0.03) and smoking to a 36% increase (*p* = 0.03). Conversely, each 10 kg increase in body weight corresponded to a 13% reduction in S100B levels (*p* = 0.008) (see Table S1).

#### 3.2.2. NSE Analysis

**Figure 3:**
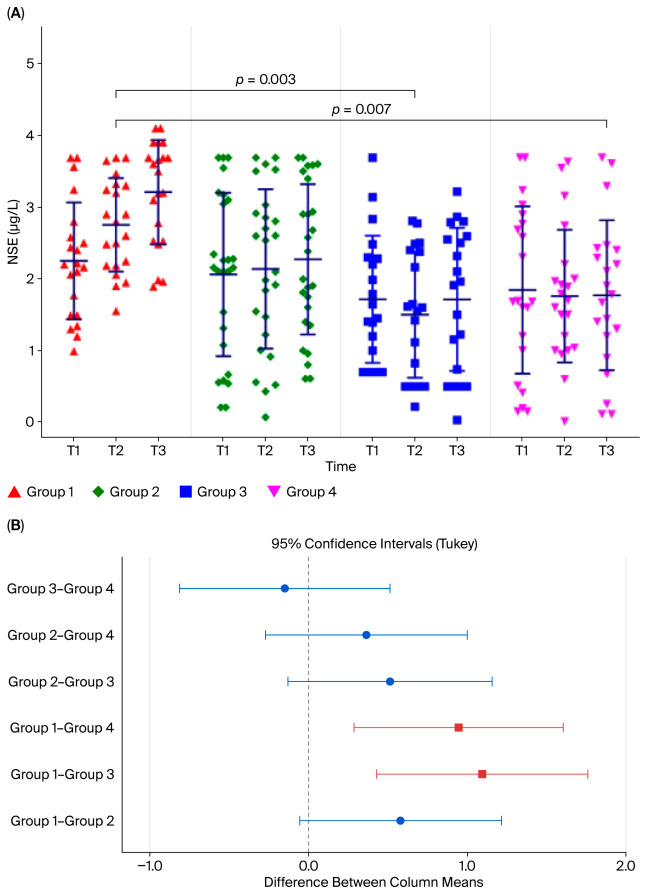
Mean (SD) neuron-specific enolase (NSE) levels (**A**) and differences between group means (**B**). Statistically significant differences are presented as red squares (**B**). Group 1: propofol/remifentanil; Group 2: propofol/remifentanil with Mg^+^/ketamine; Group 3: sevoflurane/remifentanil; and Group 4: sevoflurane/remifentanil with Mg^+^/ketamine. T1: 10 min following the onset of surgery; T2: 20 min after the initiation of controlled hypotension; and T3: upon completion of the procedure.

Participants from Group 1 (21 participants), Group 2 (28 participants), Group 3 (19 participants), and Group 4 (23 participants) each provided blood samples at three timepoints, totaling 63, 84, 57, and 69 samples, respectively, for NSE analysis. A nested one-way ANOVA group analysis (Figure 3 and Table 3) with Tukey’s multiple comparison test indicated that Group 4 exhibited significantly lower NSE values [Mean (SD): 1.79 (0.97)] than Group 1 [Mean (SD): 2.73 (0.62), *p* = 0.007]. Furthermore, Group 3 demonstrated statistically significant reductions in NSE levels [Mean (SD): 1.64 (0.76)] compared to Group 1 (*p* = 0.003). Group 2 showed comparable NSE levels [Mean (SD): 2.15 (1.00)] to the other groups. A mixed-effects model (REML) analysis followed by post hoc pairwise comparisons using the Sidak adjustment showed that Group 1 had notably higher ln NSE levels than Group 4 at both T2 (Δln = 0.90, *p* = 0.006) and T3 (Δln = 1.44, *p* < 0.001). Group 1 also displayed increased NSE levels compared to Group 3 at T2 (Δln = 1.24, *p* < 0.001) and at T3 (Δln = 1.49, *p* < 0.001). Furthermore, at T3, Group 1’s ln NSE levels were significantly higher than those of Group 2 (Δln = 0.76, *p* = 0.01). No statistically significant differences were observed between groups at T1 (NSE baseline level; see Figure S2).

Serum NSE concentrations were also evaluated on the logarithmic scale utilizing a multiple linear regression model. After controlling for gender, smoking status, and body weight, analyses revealed a significant main effect of anesthetic group on NSE concentrations (*p*= 0.001). In contrast, neither the effect of time (*p* = 0.486) nor the interaction between group and time (*p* = 0.381) reached statistical significance, indicating that changes in NSE levels over time were consistent across anesthetic protocols. Among the covariates evaluated, both age and weight were identified as independent predictors of elevated NSE concentrations (*p* = 0.012 and *p* = 0.001, respectively). ASA-PS class, gender, smoking history, surgery duration, and intraoperative hemodynamic parameters (mean HR and MAP) demonstrated no significant association with NSE levels (all *p* > 0.10). Notably, even after adjusting for these covariates, the anesthetic group remained a significant determinant of NSE concentrations (*p* = 0.001), whereas neither timepoint nor the group × timepoint interaction achieved statistical significance (Table S2).

### 3.3. Secondary Outcomes

#### 3.3.1. Recovery Time

Median verbal response and extubation times are listed in Supplementary Table S3. Post hoc Mann–Whitney tests showed that anesthesia with propofol–remifentanil (Group 1) produced consistently shorter recovery and extubation times than other protocols. Group 1 was significantly faster than Group 2 (both *p* < 0.001) and Group 4 (*p* = 0.004 for verbal response; *p* = 0.005 for extubation), while no significant difference was found between Group 1 and Group 3 (verbal response: *p* = 0.18; extubation: *p* = 0.14). Group 3 (sevoflurane–remifentanil) recovered faster than Group 2 (verbal response *p* = 0.04; extubation *p* = 0.01), while a comparison between sevoflurane regimens (Group 3 vs. Group 4) showed a non-significant trend toward quicker extubation in Group 3 (*p* = 0.05 after adjustments). All group comparisons are detailed in Table 4, and recovery times across anesthetic groups are listed in Table S3.

#### 3.3.2. Surgical Field, Bleeding, SAS, and Aldrete Score

As is customary for such procedures, blood loss was negligible and therefore not documented. Postoperative hemoglobin values remained comparable to baseline measurements. No statistically significant differences were observed among the four anesthetic groups with respect to surgical field quality, bleeding scores, sedation–agitation levels, or early post-anesthesia recovery (see Table S4). Median Likert scores for the surgical field ranged from 5 to 6 across all groups (*p* = 0.16), and bleeding scores were comparable (*p* = 0.42). The Sedation–Agitation Scale (SAS) outcomes revealed no significant variation (*p* = 0.35). Similarly, Aldrete Scores did not demonstrate clinically meaningful differences between groups (*p* = 0.16).

#### 3.3.3. Hemodynamic and Ventilatory Management

MAP was similar across all anesthetic groups at every intraoperative timepoint (T1: *p* = 0.18; T2: *p* = 0.32; T3: *p* = 0.93; see Table S5). At T2, the MAP values (mean, SD) were as follows: Group 1, 52.25 (3.28) mmHg; Group 2, 50.61 (2.84) mmHg; Group 3, 51.45 (3.33) mmHg; and Group 4, 50.80 (3.69) mmHg. HR showed no significant group differences at baseline (*p* = 0.71) or postoperatively (*p* = 0.14), with a nonsignificant intraoperative trend toward higher rates in Group 4 after adjustment (Table S6). At T2, the HR values (median, IQR) were as follows: Group 1, 56 (15) beats per minute (bpm); Group 2, 67 (13) bpm; Group 3, 58 (16) bpm; and Group 4, 65 (13) bpm. Mean PCO_2_ remained within the normal range (34–37 mmHg) throughout, with minimal and clinically irrelevant group differences (Table S7).

#### 3.3.4. Anesthetic Doses and Adjuvant Use

The dosages of propofol (mg)—averaging 697.3 (SD: 223.6) in Group 1 and 779.0 (SD: 226.9) in Group 2—as well as the Sevoflurane MAC values—minimum mean (SD) of 0.88 (0.130) and maximum mean (SD) of 1.12 (0.158) for Group 3, and minimum mean (SD) of 0.89 (0.128) and maximum mean (SD) of 1.01 (0.126) for Group 4—and remifentanil requirements all displayed normal distributions. No significant differences were observed between the groups, as detailed in Table S8. In contrast, fentanyl, clonidine, and phenylephrine doses had non-normal distributions with many zero values. The Kruskal–Wallis test results showed a significant difference in intraoperative fentanyl use (*p* = 0.03): Group 2 received more fentanyl than Group 1 (*p* = 0.02) and Group 3 (*p* = 0.01), with no other notable group differences. Clonidine and phenylephrine administration was comparable between groups, with a median dose of 0 mg, suggesting that the majority of patients did not receive either medication (Table S8).

## 4. Discussion

Induced hypotensive anesthesia is frequently used to reduce bleeding and optimize the visual field for FESS [2,3], since excessive bleeding and an inadequate surgical field during FESS can increase complications. Serious complications due to organ hypoperfusion are uncommon and morbidity resulting from major neurological complications is very low. However, cerebral ischemia is still a concern during controlled hypotension for FESS. Intraoperative hypotension, particularly its duration, is significantly associated with the occurrence of a postoperative stroke [39,40]. However, the reported safe range of arterial pressure for controlled hypotension is inconsistent and multiple risk factors are associated with complications.

The four-group design of the present study can be interpreted within a conceptual 2 × 2 factorial-like framework, defined by two key variables: the primary anesthetic regimen (propofol–remifentanil versus sevoflurane–remifentanil) and the adjunctive administration of ketamine–magnesium (present versus absent). Within this structure, comparisons between Group 1 and Group 3, as well as between Group 2 and Group 4, primarily reflect the effect of the maintenance anesthetic technique while controlling for adjunct use. Conversely, comparisons between Group 1 and Group 2, and between Group 3 and Group 4, isolate the effect of adjunctive ketamine–magnesium within each anesthetic background. In contrast, cross-factor comparisons—such as Group 2 versus Group 3—represent simultaneous variation in both factors (propofol with ketamine–magnesium versus sevoflurane without adjuncts) and therefore do not permit attribution of observed effects to a single pharmacological component. Instead, such comparisons should be interpreted as reflecting differences between composite anesthetic strategies. This distinction is essential for the interpretation of the secondary outcome findings. For example, statistically significant differences observed between Group 2 and Group 3 should not be interpreted as evidence of a superior effect of either propofol or sevoflurane alone, nor of ketamine–magnesium independently, but rather as the result of a combined pharmacological profile involving both anesthetic modality and adjunctive therapy. Accordingly, mechanistic conclusions in this study are primarily derived from within-factor comparisons, whereas cross-factor contrasts are interpreted more cautiously as reflecting clinically relevant but non-decomposable differences between multimodal anesthetic approaches.

This study provides evidence that anesthetic technique plays a critical role in modulating perioperative neuronal biomarker release among patients undergoing FESS. The findings of the present study highlight distinct and complementary patterns in the behavior of S100B and NSE, reflecting their differing biological roles in the assessment of perioperative neural stress. While both biomarkers are widely used in clinical and translational settings, they represent fundamentally different aspects of cerebral injury. S100B is primarily associated with glial activation and alterations in blood–brain barrier (BBB) permeability, whereas NSE is more specifically linked to neuronal cytoplasmic injury [22,41,42]. The findings of the present study highlight distinct and complementary patterns in the behavior of S100B and NSE, reflecting their differing biological roles in the assessment of perioperative neural stress. Accordingly, the interpretation of their perioperative dynamics requires a differentiated mechanistic approach rather than a unified interpretation.

Within this framework, the observed differences in S100B concentrations appear to be most pronounced in the group receiving sevoflurane–remifentanil combined with ketamine–magnesium (Group 4), which consistently demonstrated the lowest S100B values compared with both propofol–remifentanil (Group 1) and sevoflurane–remifentanil alone (Group 3). In contrast, no significant difference was observed between Group 1 and Group 3. This pattern suggests that the attenuation of S100B is unlikely to be attributable solely to the choice of primary anesthetic agent. Instead, it is more consistent with a combined or interaction effect, whereby the simultaneous use of sevoflurane with NMDA-modulating adjuncts such as ketamine and magnesium may enhance mechanisms related to glial stabilization and BBB integrity under conditions of controlled hypotension.

In contrast, the NSE findings demonstrate a different pattern. Both sevoflurane-based regimens (Groups 3 and 4) were associated with significantly lower NSE concentrations compared with propofol–remifentanil (Group 1), while no significant differences were observed between the two sevoflurane groups. This suggests that NSE is primarily influenced by the main effect of the anesthetic regimen, with sevoflurane providing a more consistent reduction in neuronal stress or injury markers compared with propofol-based anesthesia. The absence of additional reduction in NSE with the inclusion of ketamine–magnesium indicates that, within the conditions of the present study, adjunctive NMDA modulation does not confer a clearly additive neuroprotective effect on neuronal cytoplasmic injury beyond that achieved by sevoflurane alone.

The divergence between S100B and NSE patterns is therefore biologically meaningful rather than contradictory. The stronger and more selective reduction in S100B observed in the combined sevoflurane–ketamine–magnesium group, in the absence of a comparable incremental reduction in NSE, may indicate that these anesthetic strategies predominantly modulate perioperative glial activation and BBB-related stress, rather than directly altering the degree of neuronal injury. This interpretation is consistent with the known sensitivity of S100B to subtle, reversible disruptions in cerebral homeostasis, whereas NSE typically reflects more established neuronal cell damage [22,32,34,41]. Furthermore, the lack of significant differences in certain intergroup comparisons—such as Group 1 versus Group 3 for S100B, or Group 3 versus Group 4 for NSE—can be explained within this mechanistic framework. Specifically, these findings suggest that individual components of the anesthetic strategy may exert selective and non-uniform effects across different biological pathways, rather than producing uniform changes across all biomarkers. Consequently, comparisons that isolate only one factor (e.g., anesthetic agent alone or adjunct therapy alone) may not capture the full extent of the neurobiological response, particularly in a multifactorial perioperative environment.

Taken together, these results emphasize that anesthetic-induced neuroprotection during controlled hypotension is likely mediated through multiple, partially overlapping mechanisms. Sevoflurane appears to exert a dominant effect on neuronal stability, as reflected in NSE, whereas the combination of sevoflurane with ketamine–magnesium may provide additional modulation of glial and BBB-related processes, as reflected in S100B. This distinction underscores the importance of using multiple complementary biomarkers when evaluating perioperative neuroprotection, as reliance on a single marker may lead to incomplete or potentially misleading conclusions regarding the underlying biological effects of different anesthetic strategies.

Volatile anesthetics can induce ischemic preconditioning, preserve cerebral autoregulation, stabilize the blood–brain barrier, and reduce oxidative stress [42,43,44]. A prior study demonstrated that S100B concentrations increased during general anesthesia with propofol and remifentanil compared to baseline levels; however, these values consistently remained within the normal range [38]. Additionally, another investigation comparing propofol and desflurane anesthesia found no significant difference in S100B levels between the two anesthetic regimens [13]. Our study used a different volatile anesthetic (sevoflurane), which may be more effective during controlled hypotension, along with ketamine and magnesium, which could offer better brain protection [14,15,16,21].

Two previous experimental and translational studies indicate that volatile anesthetics like sevoflurane may provide neuroprotective benefits that depend on the dose. The first study revealed that sevoflurane at concentrations between 1.0 and 1.3 MAC has been linked to decreased cerebral injury in experimental models of ischemia–reperfusion, connecting these MAC ranges to neuroprotection [42]. In the second study, clinically relevant concentrations (approximately 1–2 MAC) have been shown to decrease cerebral metabolic rate and neuronal activity, which may contribute to neuroprotective mechanisms [44]. However, clinical evidence remains limited and somewhat inconclusive. Our study used comparable Sevoflurane MACs, indicating potential neuroprotection.

Both ketamine and magnesium exert neuroprotective effects primarily through NMDA receptor antagonism and modulation of excitotoxic pathways [45,46,47]. Ketamine has been shown to reduce neuronal apoptosis and inflammation, while magnesium attenuates calcium-mediated neuronal injury and stabilizes neuronal membranes. In addition, experimental and translational evidence suggests a potential synergistic effect when these agents are combined, due to complementary NMDA receptor blockade, particularly in conditions of controlled hypotension [17]. The neuroprotective properties of anesthetic agents, such as NMDA antagonists and volatile anesthetics, are extensively documented in the scientific literature, especially regarding their roles in ischemia–reperfusion models and perioperative cerebral protection [48].

Patient-related factors also emerged as important predictors: age and weight independently correlated with elevated NSE levels. Such relationships are consistent with the literature reporting age-dependent increases in neuronal vulnerability and biomarker release, as well as metabolic and inflammatory alterations associated with higher body mass [10]. In contrast, no significant associations were observed with ASA class, smoking status, intraoperative hemodynamics, or surgical duration. These results imply that intrinsic biological characteristics may exert a stronger influence on perioperative neuronal biomarker dynamics than moderate variations in physiological parameters during anesthesia.

Despite the clear biomarker differences across anesthetic techniques, these differences did not translate into differences in immediate clinical recovery or operative conditions. Surgical field quality, bleeding scores, sedation–agitation levels, and Aldrete Scores remained comparable among groups, consistent with prior evidence that biochemical indicators of neuronal stress do not necessarily correlate with intraoperative clinical metrics [7]. The only clinically significant divergence was observed in recovery times: propofol–remifentanil without adjuvants produced the fastest emergence profile, aligning with the well-characterized pharmacokinetic advantages of propofol-based anesthesia [8]. Conversely, the addition of ketamine–magnesium—particularly in propofol-based regimens—prolonged time to verbal response and extubation, supporting existing data that NMDA antagonists can delay emergence [9].

In light of the potential adverse effects associated with magnesium, it is important to note that magnesium infusion can result in delayed emergence, enhanced neuromuscular blockade, and hypotension, particularly at elevated doses or in patients with heightened vulnerability. Nonetheless, in the present study, magnesium was administered within clinically established parameters, and neither significant recovery delays nor notable neuromuscular complications were detected.

Our study is limited by its single-center design and small, uneven group sizes. Nonetheless, each group included at least 20 patients based on power analysis. Additionally, baseline biomarker concentrations were measured 10 min after the onset of surgery, during which all patients received propofol for anesthesia induction. This approach may introduce bias, as it does not adequately highlight the potential protective effects of sevoflurane. However, the interval between anesthesia induction and the initiation of surgery was comparable across all groups (ranging from 7 to 10 min), during which time patients received their assigned medications. Conversely, the groups were carefully structured to identify distinctions in neuroprotection between propofol and sevoflurane, while also accounting for the neuroprotective effects of magnesium and ketamine. A notable limitation of the present study is that the relative contributions of ketamine and magnesium to the observed effects cannot be definitively delineated. As both agents were co-administered in the relevant study groups, it is not possible to isolate their individual effects or to determine whether the observed outcomes are attributable primarily to one agent or to a potential synergistic interaction between them. This limitation should be considered when interpreting the study findings, particularly in relation to the mechanistic pathways underlying the observed physiological and biomarker changes. Future investigations employing study designs that allow for independent and combined evaluation of these agents—such as factorial or multi-arm comparative trials—would be valuable in clarifying their respective roles and potential interactions, thereby providing more precise mechanistic insight and guiding optimized anesthetic strategies. Furthermore, some variables were not fully considered, such as the involvement of different surgeons and variations in disease severity, both of which could influence outcomes like surgery duration and blood loss. In our study, the surgical team remained consistent, and the duration of surgery was comparable between the groups (Table 1). Our study also did not examine the long-term effects on brain or cognitive function. Finally, although randomization was implemented, the study was not double-blinded due to safety reasons, which could potentially introduce observer bias. Specifically, the attending anesthesiologist and the nursing staff in the operating room were informed about the medication used, whereas the postoperative outcome assessors, the statistician, as well as the individual measuring the levels of biomarkers remained blinded to the group designations.

To our knowledge, this is the first study comparing propofol and sevoflurane with adjuncts, using two neuroprotection biomarkers. The results suggest that anesthetic choice should be tailored to neurological risk and recovery needs.

## 5. Conclusions

Taken together, these findings underscore anesthetic selection as a modifiable determinant of perioperative neuroprotection. Sevoflurane-based regimens and targeted Mg and NMDA receptor modulation may optimize neuronal preservation during FESS, although these biochemical benefits must be weighed against differences in emergence profiles. Future studies integrating serial biomarker measurements with cognitive testing and neuroimaging are needed to clarify the clinical significance of these perioperative neuronal changes and to guide personalized anesthetic strategies in populations at increased risk of postoperative neurocognitive impairment.

## Figures and Tables

**Table 1:** Patients’ demographic and baseline characteristics.

Variable	Group 1 *n* [%] Mean [SD]	Group 2 *n* [%] Mean [SD]	Group 3 *n* [%] Mean [SD]	Group 4 *n* [%] Mean [SD]	*p*-Value
Gender					0.64
Male	12 [57.0]	20 [71.4]	12 [60]	15 [71.4]	
Female	9 [42.9]	6 [28.6]	8 [40]	6 [28.6]	
Age (years)	43.047 [18.94]	40.53 [13.39]	44.47 [13.41]	43.23 [17.38]	0.85
Weight (kg)	77.19 [17.48]	81.17 [15.79]	84.45 [18.3]	81.19 [17.07]	0.6
BMI (kg/m^2^)	25.76 [3.7]	27.18 [4.13]	28.31 [5.69]	27.27 [5.41]	0.43
ASA					0.37
1	11 [55]	15 [53.6]	11 [61.1]	8 [38.1]	
2	8 [40]	13 [46.4]	5 [27.8]	10 [47.6]	
3	1 [5]	0	2 [11.1]	3 [14.3]	
Smoking status					0.2
Smoker	8 [40]	15 [53.6]	4 [23.5]	12 [57.1]	
Non-smoker	12 [60]	13 [46.4]	13 [76.5]	9 [42.9]	
Duration of the surgery (min)	98.4 [28.6]	103.85 [21.9]	99.52 [29.17]	114.23 [34.13]	0.43

Demographic parameters (gender, age, weight, and BMI), ASA physical status, smoking status, and surgical duration for patients allocated to the four anesthetic groups. Continuous variables are presented as mean ± SD. Categorical variables are expressed as *n* (%). *p*-values refer to comparisons across all four groups and were calculated using ANOVA for normally distributed variables, Kruskal–Wallis tests for non-normally distributed variables, and χ^2^ tests for categorical data. No statistically significant differences were observed among groups, indicating appropriate baseline comparability. Group 1: propofol/remifentanil; Group 2: propofol/remifentanil with Mg^+^/ketamine; Group 3: sevoflurane/remifentanil; and Group 4: sevoflurane/remifentanil with Mg^+^/ketamine.

**Table 2:** Differences in S100B levels between groups.

S100B—Comparisons Between Groups			
Tukey’s Multiple Comparisons Test	Mean Diff.	95% CI of Diff.	Adjusted *p*-Value
Group 1 vs. Group 2	0.22	−0.38 to 0.82	0.66
Group 1 vs. Group 3	0.15	−0.47 to 0.78	0.85
Group 1 vs. Group 4	0.79	0.18 to 1.40	0.01
Group 2 vs. Group 3	−0.06	−0.67 to 0.54	0.98
Group 2 vs. Group 4	0.57	−0.02 to 1.20	0.06
Group 3 vs. Group 4	0.64	0.02 to 1.30	0.04

Pairwise post hoc comparisons of S100B concentrations between the four anesthetic groups based on the mixed-effects model using log-transformed values. Results are presented as mean differences in log scale (Δln), 95% Confidence Intervals, and Tukey’s adjusted *p*-values. Group 1: propofol/remifentanil; Group 2: propofol/remifentanil with Mg^+^/ketamine; Group 3: sevoflurane/remifentanil; and Group 4: sevoflurane/remifentanil with Mg^+^/ketamine.

**Table 3:** Differences in NSE levels between groups.

Neuron-Specific Enolase (NSE)—Comparisons Between Groups			
Tukey’s Multiple Comparisons Test	Mean Diff.	95% CI of Diff.	Adjusted *p*-Value
Group 1 vs. Group 2	0.58	−0.05 to 1.21	0.07
Group 1 vs. Group 3	1.09	0.43 to 1.76	0.003
Group 1 vs. Group 4	0.94	0.28 to 1.60	0.007
Group 2 vs. Group 3	0.51	−0.12 to 1.15	0.12
Group 2 vs. Group 4	0.36	−0.27 to 1.00	0.32
Group 3 vs. Group 4	−0.15	−0.81 to 0.51	0.88

Pairwise post hoc comparisons of neuron-specific enolase (NSE) concentrations between the four anesthetic groups based on the mixed-effects model using log-transformed values. Results are presented as mean differences in log scale (Δln), 95% Confidence Intervals, and Tukey’s adjusted *p*-values. Group 1: propofol/remifentanil; Group 2: propofol/remifentanil with Mg^+^/ketamine; Group 3: sevoflurane/remifentanil; and Group 4: sevoflurane/remifentanil with Mg^+^/ketamine.

**Table 4:** Pairwise Mann–Whitney U comparisons for recovery and extubation time between groups (time to verbal response and extubation time).

**Recovery Time (Time to Verbal Response, min)**	**U**	**Z**	***p*** **-Value**
Group 1 vs. Group 2	113.5	−3.49	<0.001
Group 1 vs. Group 3	126.0	−1.34	0.18
Group 1 vs. Group 4	100.0	−2.88	0.004
Group 2 vs. Group 3	151.0	−2.04	0.04
Group 2 vs. Group 4	290.0	−0.08	0.93
Group 3 vs. Group 4	122.5	−1.65	0.10
**Extubation Time (min)**	**U**	**Z**	***p*** **-Value**
Group 1 vs. Group 2	115.5	−3.44	<0.001
Group 1 vs. Group 3	122.0	−1.46	0.14
Group 1 vs. Group 4	102.5	−2.81	0.005
Group 2 vs. Group 3	128.5	−2.57	0.01
Group 2 vs. Group 4	280.0	−0.28	0.77
Group 3 vs. Group 4	112.0	−1.95	0.05

Pairwise post hoc comparisons of recovery time (time to verbal response and extubation time) between the four anesthetic groups using the Mann–Whitney U test. Results are reported as U statistic, Z score, and two-tailed *p*-values. Significant differences (*p* < 0.05) indicate slower recovery in Group 2 compared with Group 1 and Group 3, and slower recovery in Group 4 compared with Group 1. Significant findings indicate longer extubation times in Group 2 compared with Groups 1 and 3, and in Group 4 compared with Group 1. A borderline trend was observed between Groups 3 and 4 (*p* = 0.05). Group 1: propofol/remifentanil; Group 2: propofol/remifentanil with Mg^+^/ketamine; Group 3: sevoflurane/remifentanil; and Group 4: sevoflurane/remifentanil with Mg^+^/ketamine.

## Data Availability

The data supporting this study are available from the corresponding author upon reasonable request. The data are not publicly accessible due to privacy and ethical considerations.

## Supplementary Materials

The following supporting information can be downloaded at: https://www.mdpi.com/article/10.3390/medicina62061006/s1, Figure S1: S100B Post hoc pairwise comparisons adjusted by the Sidak method, Between Groups and at different timepoints; Figure S2: Neuron specific enolase (NSE) Post hoc pairwise comparisons adjusted by the Sidak method, Between Groups and at different timepoints; Table S1: Fixed-Effects Results of the Mixed-Effects Model for S100B (Log-Transformed Values); Table S2: Fixed-Effects Results of the Mixed-Effects Model for NSE (Log-Transformed Values); Table S3: Recovery Times Across Anesthetic Groups; Table S4: Secondary Intraoperative and Early Recovery Outcomes (Surgical Field, Bleeding, SAS, Aldrete Score); Table S5: Mean Arterial Pressure (MAP) Across Anesthetic Groups at Three Timepoints; Table S6: Heart Rate (HR) Across Anesthetic Groups at Three Timepoints; Table S7: Arterial Carbon Dioxide (PaCO_2_) Levels Across Anesthetic Groups; Table S8: Intraoperative Anesthetic Drug Requirements across Groups.

## Abbreviations

The following abbreviations are used in this manuscript:

ANOVA	Analysis of Variance
ASA	American Society of Anesthesiologists
BBB	Blood–Brain Barrier
BIS	Bispectral index
BMI	Body mass index
CI	Confidence interval
CNS	Central Neuron System
ETCO_2_	End-tidal carbon dioxide
FESS	Functional endoscopic sinus surgery
HR	Heart rate
IV	Intravenous
LOD	Limit of detection
MAC	Minimum alveolar concentration
MAP	Mean arterial pressure
Mg^+^	Magnesium sulfate
NSE	Neuron-specific enolase
NMDA	N-Methyl-D-aspartate
PACU	Post-anesthesia care unit
PCO_2_	Partial pressure of carbon dioxide
REML	Restricted maximum likelihood
S-100B	S-100 beta protein
SAS	Sedation–Agitation Scale
SD	Standard deviation
