# Supplementary material for: Variations in S-100Β and Neuron-Specific Enolase Levels During Functional Endoscopic Sinus Surgery Under Moderately Controlled Hypotension Using Four Distinct Anesthetic Protocols: A Randomized Controlled Study
Source: Medicina (Kaunas). 2026 May 22;62(6):1006. doi: 10.3390/medicina62061006 (PMC13304107; doi:10.3390/medicina62061006)
Supplement: Supplementary file 1 [file medicina-62-01006-s001.zip › medicina-4275335-supplementary.pdf]

Supplementary material

Figure S1: S100B Post-hoc pairwise comparisons adjusted by the Sidak method, Between Groups and at different timepoints.

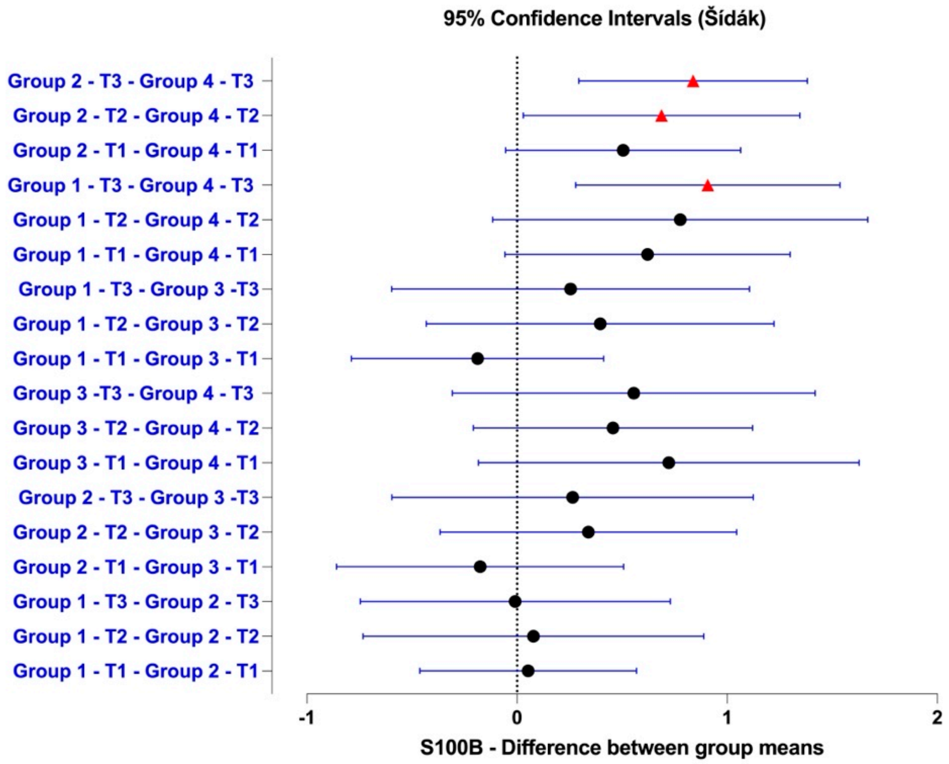

Table S1. Fixed-Effects Results of the Mixed-Effects Model for S100B (Log-Transformed Values)

Effect	Numerator df	Denominator df	F	p-value
**Group**	3	80	3.297	**0.025**
**Timepoint**	2	86	3.968	**0.022**
**Group × Timepoint**	6	85	0.704	0.648
**Gender**	1	80	4.663	0.034
**Smoking**	1	80	4.682	0.033
**Weight**	1	80	7.507	0.008

Fixed-effects results from the mixed-effects model evaluating the impact of anesthetic group, timepoint, and their interaction on log-transformed S100B levels. Both group and timepoint showed statistically significant effects, indicating differences in S100B profiles between anesthetic regimens and across perioperative timepoints. The GROUP × timepoint interaction was not significant, suggesting that temporal changes in S100B were similar across the four anesthetic groups.

Figure S2: Neuron specific enolase (NSE) Post-hoc pairwise comparisons adjusted by the Sidak method, Between Groups and at different timepoints.

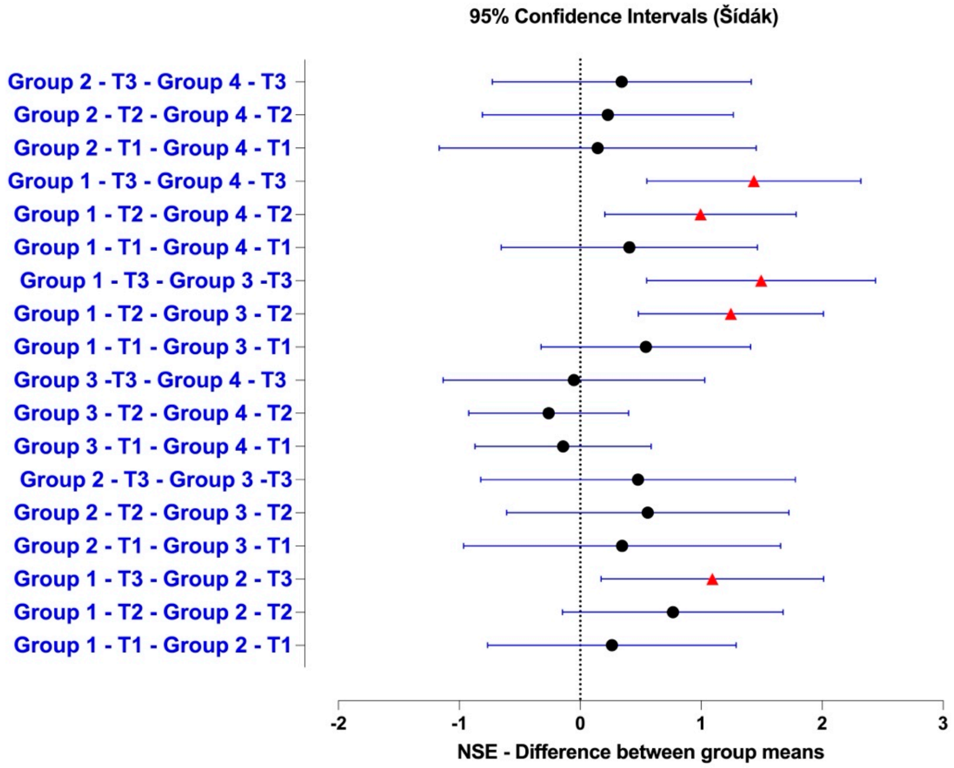

Table S2. Fixed-Effects Results of the Mixed-Effects Model for NSE (Log-Transformed Values)

	**Numerator df**	**Denominator df**	**F-value**	**p-value**
**Intercept**	1	49.508	4.612	0.037
**Group**	3	51.968	6.002	0.001
**Timepoint**	2	58.529	0.731	0.486
**Timepoint × Group**	6	58.459	1.087	0.381
**ASA**	1	49.613	1.497	0.227
**Gender**	1	49.564	0.931	0.339
**Smoking**	1	49.738	1.893	0.175
**Surgery duration (min)**	1	49.487	2.208	0.144
**Weight**	1	49.895	11.650	0.001
**Age**	1	49.516	6.865	0.012
**HR_mean**	1	49.638	2.501	0.120
**MAP_mean**	1	49.514	0.864	0.357

Fixed-effects estimates from the mixed-effects model evaluating the association between anesthetic group, timepoint, and clinical covariates with log-transformed NSE concentrations. The table reports numerator and denominator degrees of freedom, F-statistics, and p-values for each fixed effect. A significant main effect of GROUP (p = 0.001) indicates that anesthetic regimen was an independent determinant of NSE levels. Timepoint and the Timepoint × GROUP interaction was not significant. Among covariates, weight (p

= 0.001) and age (p = 0.012) were significant predictors of higher NSE concentrations, while ASA class, gender, smoking status, surgical duration, HR_mean, and MAP_mean were not associated with NSE. Model estimates are adjusted for all listed variables.

Table S3. Recovery Times Across Anesthetic Groups

Recovery Time Median (IQR)	Group 1	Group 2	Group 3	Group 4	p-value
**Time to verbal response (min)**	8.0 (3.88)	13.0 (8.75)	11.0 (5.50)	13.0 (10.50)**	0.002
**Extubation time (min)**	8.0 (5.39)	13.0 (5.86)	11.0 (4.50)	13.0 (9.25)	<0.001

Recovery metrics expressed as median (IQR) for time to verbal response and extubation time across the four anesthetic groups. Group 1 demonstrated the shortest recovery times for both outcomes, while Groups 2 and 4 showed prolonged emergence. Group 3 exhibited intermediate recovery characteristics. Statistical comparisons were performed using the Mann–Whitney U test.

Table S4. Secondary Intraoperative and Early Recovery Outcomes (Surgical Field, Bleeding, SAS, Aldrete Score)

Secondary outcomes Median (IQR)	Group 1	Group 2	Group 3	Group 4	p-value
**Surgical field (Likert 1–5)**	5 (4–6)	5 (4–6)	6 (5–6.5)	6 (5–7)	0.16
**Bleeding score (1–5)**	3 (2–4)	2 (1–3)	2 (1–3)	2 (1–3)	0.42
**SAS (Sedation–Agitation Score)**	3.5 (3–4)	3 (3–4)	4 (3–4.5)	3 (3–4)	0.35
**Aldrete score**	9 (8–10)	9 (8–11)	8 (7–9)	8 (7–9)	0.16

Secondary intraoperative and postoperative recovery outcomes across the four anesthetic groups. Values are expressed as median (IQR). Comparisons were performed using the Kruskal–Wallis test. No statistically significant differences were observed between groups for any of the secondary outcomes, indicating comparable surgical conditions and early recovery quality across anesthetic regimens.

Table S5. Mean Arterial Pressure (MAP) Across Anesthetic Groups at Three Timepoints

MAP (mmHg) – Mean (SD)	Group 1	Group 2	Group 3	Group 4	p-value
**MAP T1 (baseline)**	89.0 (9.6)	96.1 (11.8)	93.3 (11.3)	93.4 (11.0)	0.18
**MAP T2 (intraoperative)**	52.25 (3.28)	50.61 (2.84)	51.45 (3.33)	50.80 (3.69)	0.32
**MAP T3 (recovery)**	89.1 (15.6)	88.9 (16.1)	88.4 (16.2)	86.8 (12.8)	0.93

Mean arterial pressure (MAP) recorded at T1:10 minutes following the onset of surgery, T2: 20 minutes after the initiation of controlled hypotension, T3: upon completion of the procedure. Values are presented as mean ± SD. Comparisons across the four anesthetic groups were performed using ANOVA. No statistically significant differences were observed at any timepoint, suggesting similar hemodynamic profiles among groups during the perioperative period.

Table S6. Heart Rate (HR) Across **Anesthetic Groups** at Three Timepoints

**Heart Rate (bpm) Median - IQR**	**Group 1**	**Group 2**	**Group 3**	**Group 4**	**p-value**
**HR T1**	80 [36]	83 [21]	82 [23]	82 [25]	0.71
**HR T2**	56 [15]	67 [13]	58 [16]	65 [13]	0.05
**HR T3**	81 [30]	75 [16]	82 [18]	80 [20]	0.14

Heart rate (HR) values at T1:10 minutes following the onset of surgery, T2: 20 minutes after the initiation of controlled hypotension, T3: upon completion of the procedure. Values are presented as median [IQR] and compared across groups using the Kruskal–Wallis test. A borderline difference was observed intraoperatively (p = 0.050), with Group 2 showing higher HR compared with the other groups. No significant differences were observed at T1 or T3

Table S7. Arterial Carbon Dioxide ($PaCO_2$) Levels Across Anesthetic Groups

**$PaCO_2$ (mmHg)**	**Group 1**	**Group 2**	**Group 3**	**Group 4**	**p-value**
**$PaCO_2$ T1**	36.4 (4.3)	37.5 (3.7)	37.4 (4.4)	35.0 (3.7)	0.26
**$PaCO_2$ T2**	36.2 (4.1)	36.6 (3.4)	35.5 (3.0)	34.9 (4.2)	0.36
**$PaCO_2$ T3**	35.0 (3.0)	35.5 (4.0)	35.6 (3.0)	35.6 (3.5)	0.70

Arterial carbon dioxide tension ($PaCO_2$) measured at T1:10 minutes following the onset of surgery, T2: 20 minutes after the initiation of controlled hypotension, T3: upon completion of the procedure. Values expressed as mean ± SD and compared across groups using ANOVA. No statistically significant differences were observed at any timepoint, indicating comparable ventilatory and respiratory management across anesthetic techniques.

Table S8. Intraoperative Anesthetic Drug Requirements across Groups

**Variable**	**Group 1**	**Group 2**	**Group 3**	**Group 4**	**p-value**
**Propofol (mg), Mean (SD)**	697.3 (223.6)	779.0 (226.9)	—	—	0.954
**MAC min, Mean (SD)**	—	—	0.88 (0.130)	0.89 (0.128)	0.67
**MAC max, Mean (SD)**	—	—	1.12 (0.158)	1.01 (0.126)	0.50
**Remifentanil (μg), Mean (SD)**	1150 (617.6)	1506.14 (627.65)	1100.36 (819.7)	1070.6 (714.26)	0.192
**Additive- Median [IQR]**					
**Fentanyl (μg)**	**250 [50]**	**300 [200]**	**250 [200]**	**250 [150]**	**0.032**
**Clonidine (μg)**	0 [137.5]	0 [150]	0 [100]	0 [150]	0.46
**Phenylephrine (mg)**	0 [0.2]	0 [0.08]	0 [0.18]	0 [0.14]	0.31

Intraoperative anesthetic dosing across the four anesthetic groups. Propofol, remifentanil, and MAC values are presented as mean ± SD and compared using ANOVA. Fentanyl, clonidine, and phenylephrine doses were non-normally distributed and are presented as median [IQR], compared using the Kruskal–Wallis test. A significant difference was observed only in intraoperative fentanyl administration (p = 0.032), with Group 2 receiving higher doses compared with Groups 1 and 3 on pairwise analysis.
